# Comprehensive Analysis of the Brain-Expressed X-Link Protein Family in Glioblastoma Multiforme

**DOI:** 10.3389/fonc.2022.911942

**Published:** 2022-07-04

**Authors:** Adilai Aisa, Yinuo Tan, Xinyu Li, Ding Zhang, Yun Shi, Ying Yuan

**Affiliations:** ^1^ Department of Medical Oncology, Key Laboratory of Cancer Prevention and Intervention, Ministry of Education, The Second Affiliated Hospital, Zhejiang University School of Medicine, Hangzhou, China; ^2^ Cancer Center, Zhejiang University, Hangzhou, China; ^3^ Nursing Department, The Second Affiliated Hospital, Zhejiang University School of Medicine, Hangzhou, China

**Keywords:** glioblastoma, immunomodulation, prognosis, BEX family, TCEAL family

## Abstract

Glioblastoma multiforme (GBM) is the most common, malignant, and deadly primary brain tumor in adults. Brain-expressed X-link (BEX) protein family is involved in tumorigenesis. Here, we have explored the biological function and the prognostic value of the BEX family in GBM. Differentially expressed BEX genes between GBM and normal tissue were screened by using The Cancer Genome Atlas (TCGA) database. Univariate and multivariate Cox regression analyses identified the prognosis‐related genes BEX1, BEX2, and BEX4, which were involved in the regulation of immune response. The results of correlation analysis and protein–protein interaction network (PPI network) showed that there was a significant correlation between the BEX family and TCEAL family in GBM. Furthermore, the expression of transcription elongation factor A (SII)-like (TCEAL) family is generally decreased in GBM and related to poor prognosis. With the use of the least absolute shrinkage and selection operator (LASSO) Cox regression, a prognostic model including the BEX family and TCEAL family was built to accurately predict the likelihood of overall survival (OS) in GBM patients. Therefore, we demonstrated that the BEX family and TCEAL family possessed great potential as therapeutic targets and prognostic biomarkers in GBM. Further investigations in large‐scale, multicenter, and prospective clinical cohorts are needed to confirm the prognostic model developed in our study.

## Introduction

Glioblastoma multiforme (GBM) is a primary malignant glioma. It is one of the most common malignant brain tumors with high invasiveness and lethality (1). The general manifestations of GBM patients are headache, dizziness, nausea, convulsions, hemiplegia, memory disorders, or personality changes, of which convulsions are the most common clinical symptoms (2). CT is the main diagnostic method at present (3). The risk factors for GBM remain uncertain. Male gender, age group over 70, and Caucasian race seem to be independent prognostic factors for GBM (4). At present, the treatment of GBM includes tumor resection, radiotherapy, and temozolomide adjuvant chemotherapy (5). Recently, immunotherapy has also shown some efficacy in the treatment of GBM (6). However, due to high drug resistance and close to 100% recurrence rate, the prognosis of GBM patients is poor, and the median survival time is only about 15 months (7). Therefore, in order to improve the efficacy of GBM, reduce drug resistance and recurrence rate, prolong the life of GBM patients, and improve their survival and treatment, further research on the mechanism of GBM occurrence and development is the main challenge at present.

The BEX family consists of five members, BEX1–5, located on the Xq22 chromosome. Members of the BEX family are widely expressed in several types of tissues and are closely associated with transcriptional regulation and signaling pathways, including neurodegeneration, cell cycle, apoptosis, autophagy, and tumor growth (8–10). The aforementioned studies highlighted an association between the BEX family members and GBM (11–13). However, there are few studies on the functional mechanism and therapeutic significance of the whole BEX family in GBM.

In this study, by comprehensively analyzing RNA‐seq profiles and clinical information of GBM in The Cancer Genome Atlas (TCGA) database, we explored BEX family expression features and potential biological functions in GBM and firstly reported the correlation between the BEX family and TCEAL family in GBM. In addition, we built a prognostic model including the BEX family and TCEAL family to accurately predict the likelihood of overall survival (OS) in GBM patients.

## Material and Methods

### Data Retrieval and Processing for This Study

The RNA‐seq profiles and clinical data of GBM patients from TCGA GBM cohort database (https://portal.gdc.cancer.gov/) were collected (14). The expression of the BEX family in GBM was analyzed and visualized on the online tool (http://gepia.cancer-pku.cn/). The data on WHO classification, IDH mutation status, and 1p/19q deletion are from the study of Ceccarelli et al. (15).

### Correlation and Functional Enrichment Analysis of BEX Family Genes

The expression profiles (HTSeq-FPKM) between the high BEX expression group and the low BEX expression group were compared to identify differentially expressed genes (DEGs) using Wilcoxon rank-sum test in the R language-related software, stat package (3.6.3). Differences with a |log fold change (FC)| > 1.5 and an adjusted p-value <0.05 were considered as threshold values for identifying DEGs.

The Kyoto Encyclopedia of Genes and Genomes (KEGG) database was used to assign biological functions and utilities of target genes. R package Clusterprofiler and GOplot were applied to perform GOKEGG function enrichment analyses on the DEGs identified between the high and low BEX expression groups (16, 17). The default parameters in the tool were used, and enriched pathways were ranked according to their enrichment scores. A p-value of <0.05 was identified as enriched functions.

The Search Tool for the Retrieval of Interacting Genes (STRING; http://string-db.org; version 10.0) online database was used to predict the protein–protein interaction network of the BEX family co-expressed genes in GBM and to analyze the functional interactions among proteins. An interaction with a combined score >0.4 was considered statistically significant.

### Gene Set Enrichment Analysis

Gene Set Enrichment Analysis (GSEA) is a computational method that determines whether a defined set of genes exhibits statistically significant concordant differences between two biological states (18). The gene set database used in this study is MSigDB Collections (https://www.gsea-msigdb.org/gsea/msigdb/index.jsp). The analysis and visualization of GSEA were performed with the R package ClusterProfiler (16) to elucidate the significant function and pathway differences between the high and low BEX expression groups. Each analysis procedure was repeated 1,000 times. A function or pathway term with an adjusted p-value <0.05 and a false discovery rate (FDR) <0.25 was considered to be statistically significant enrichment.

### Immune Infiltrate Analysis

The correlation between BEX family genes and the abundance of immune infiltrates, including B cells, CD4+ T cells, CD8+ T cells, neutrophils, macrophages, and dendritic cells, were downloaded *via* tumor immune estimation resource (19) (TIMER; https://cistrome shinyapps.io/timer/). In addition, based on expression data, the immune score, stromal score, and ESTIMATE score of each GBM patient were analyzed by R packages “GSVA packages” to access the infiltration level of immune cells and the level of stromal cells in tumor tissues (20). Subsequently, Pearson’s correlation coefficient was calculated to evaluate the associations between the expression of prognosis‐related genes and the above three scores.

### Construction and Evaluation of the Nomogram and Prognostic Model

To individualize the predicted survival probability for l, 3, and 5 years, a nomogram was constructed based on the results of the multivariate analysis. The RMS R package was used to generate a nomogram including clinical characteristics significantly associated with the BEX family and calibration plots. Calibration and discrimination are the most used methods for evaluating the performance of models (21). In this study, the calibration curves were graphically assessed by mapping the nomogram-predicted probabilities against the observed rates, and the 45° line represented the best predictive values. The concordance index (C-index) was used to determine the discrimination of the nomogram, which was calculated by a bootstrap approach with 1,000 resamples. In addition, using the least absolute shrinkage and selection operator (LASSO) Cox regression, a prognostic model including the BEX family and TCEAL family was built to accurately predict the likelihood of l, 3, and 5 years OS in GBM patients. All statistical tests were two-tailed with the statistical significance level set at 0.05.

### Statistical Analysis

All statistical analyses and plots were conducted using R (version 3.6.3). Wilcoxon rank-sum test was used to analyze the expression of the BEX family in non-paired samples. The Kruskal–Wallis test, Dunn’s test, Wilcoxon rank-sum test, and logistic regression evaluated relationships between clinical-pathologic features and BEX family expression. Furthermore, receiver operating characteristic (ROC) analysis and the frequently used method for binary assessment were performed using the pROC package to assess the effectiveness of expression to GBM from normal samples. The computed area under the curve (AUC) value ranging from 0.5 to 0.1 indicates the discriminative potential from 50% to 100%. The prognostic data were obtained from *Cell* (22). In this study, it was assumed that the hazard rates of any two individuals were proportional, and based on this assumption, Cox’s regression model was used. The Kaplan–Meier method was used to evaluate prognostic factors; in all tests, a p-value <0.05 was considered statistically significant.

## Results

### Expression Level of BEX Family in Glioblastoma Multiforme

**Figure 1:**
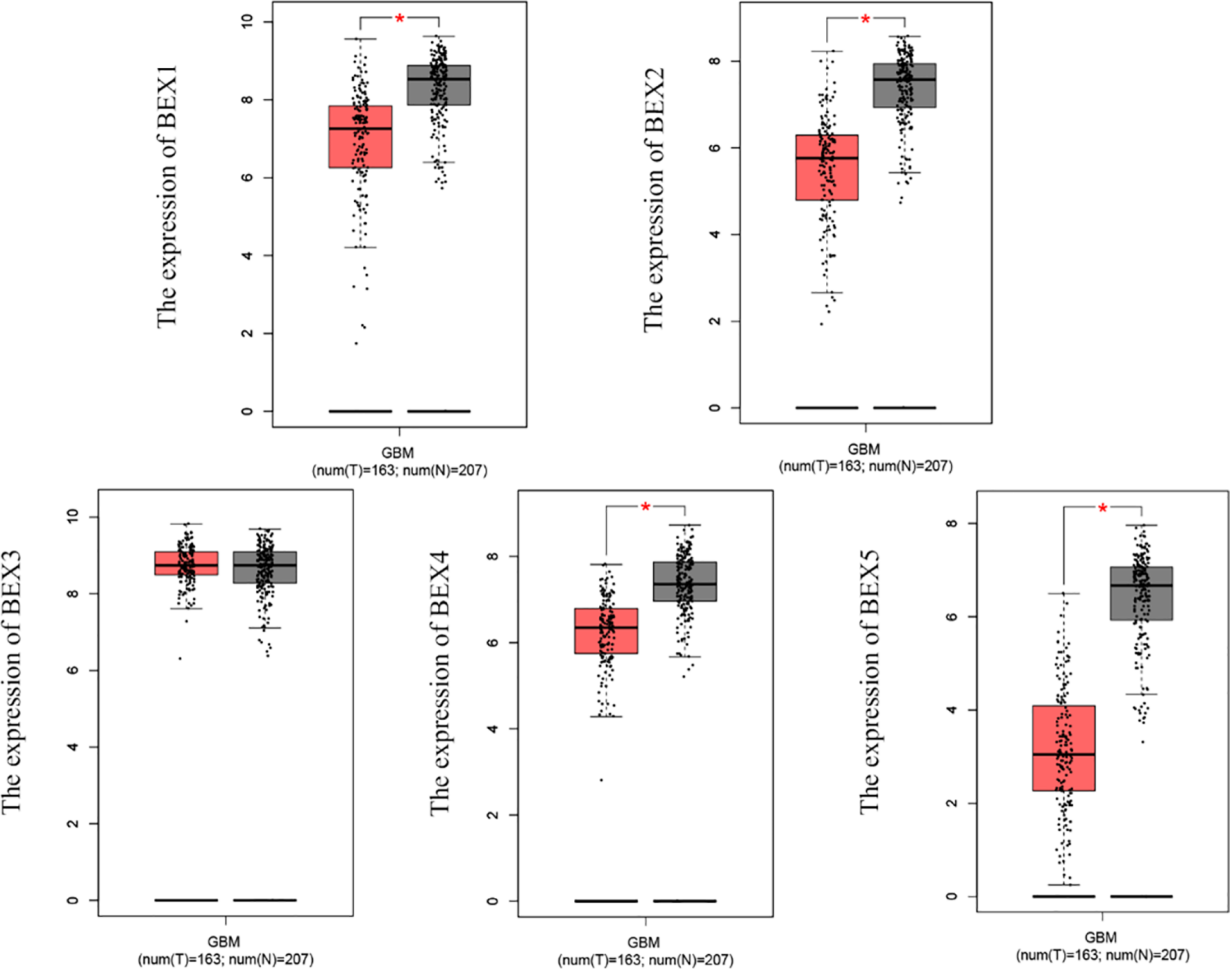
The mRNA expression of BEX family in GBM based on TCGA database and GTEx database. GBM, glioblastoma multiforme; TCGA, The Cancer Genome Atlas; GTEx, Genotype-Tissue Expression ns, p≥0.05; *p< 0.05; **p<0.01; ***p<0.001.

According to the matched analysis of 163 GBM cases in TCGA database and 207 normal tissues in Genotype-Tissue Expression (GTEx) and TCGA normal database, the expression of mRNA of BEX family members was significantly downregulated in GBM, except BEX3. The results of the Mann–Whitney U test (Wilcoxon rank-sum test) showed that the difference between tumor and normal groups was statistically significant (p < 0.05) (**Figure 1**).

### The Relationship Between BEX Family Expression and Clinical Indicators in Glioblastoma Multiforme Patients

We next compared BEX family expression among groups of patients through TCGA database, according to different clinical indicators. According to their malignancy, gliomas have been classified in four grades by WHO, and the higher grade was more malignant and related to a worse prognosis than the lower grade (23). Regarding the WHO grade of GBM, the expression of the BEX family was significantly higher in the grade II (G2) group than in G3 and G4 groups (G2>G3>G4) (**Supplementary Figure 1A** and **Supplementary Table 1**). IDH status is another new classification of GBM and divides it into three subgroups based on isocitrate dehydrogenase (IDH) mutations: IDH wild type, IDH mutant, and not otherwise specified (NOS). According to the previous reports, the prognosis of GBM patients with IDH mutant is better than that of wild type (5). Our study revealed that BEX family gene expression of GBM tended to be downregulated in the IDH wild-type group than in the IDH mutant group (except BEX5) (**Supplementary Figure 1B** and **Supplementary Table 1**). Based on the new WHO integrated diagnosis, 1p/19q codeletion status reflects the genetic profile of GBM, and there is evidence that the presence of 1p/19q codeletion is not only a positive prognostic indicator but also a strong predictor of chemosensitivity (24). In this study, we found that 1p/19q codeletion status was associated with higher expression of the BEX family (**Supplementary Figure 1C** and **Supplementary Table 1**). GBM patients with different histological types also showed a different level of BEX family expression (**Supplementary Figure 1D** and **Supplementary Table 1**). We found that BEXs were obviously decreased in the glioblastoma subtype with respect to other subtypes.

### Identification of Prognosis‐Related BEXs in Glioblastoma Multiforme Samples

**Figure 2:**
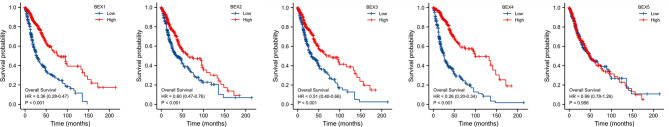
The OS of BEX family for GBM patients. The prognostic value of BEX family mRNA expression for OS in GBM patients by Kaplan–Meier analysis. The Cox regression analysis was performed to evaluate survival differences with the best cutoff value. OS, overall survival; GBM, glioblastoma multiforme.

After univariate Cox regression analysis, we found that BEX1~4 had a significant correlation with GBM (**Table 1**). In addition, the Kaplan–Meier survival curve based on the median expression value showed that the high expression group of BEX1, BEX2,BEX3, and BEX4 had a better prognosis than the low expression group in the OS aspects (log-rank p-score <0.001), while bex5 had no significant prognostic value (**Figure 2**). Subsequently, multivariate Cox regression analysis indicated that BEX1 (hazard ratio [HR]: 0.5; p < 0.001), BEX2 (HR: 1.38; p = 0.029), and BEX4 (HR: 0.2; p < 0.001) exhibited an independent prognostic value for GBM (**Table 1**).

**Table 1:** Univariate and multivariate Cox regression analyses of BEX family in GBM.

Characteristics	Total (N)	Univariate analysis		Multivariate analysis	
		Hazard ratio (95% CI)	p-Value	Hazard ratio (95% CI)	P-value
BEX2	695				
Low	347	Reference			
High	348	0.596 (0.467–0.761)	**<0.001**	1.380 (1.034–1.842)	**0.029**
BEX3	695				
Low	347	Reference			
High	348	0.513 (0.401–0.657)	**<0.001**	1.243 (0.912–1.693)	0.168
BEX4	695				
Low	348	Reference			
High	347	0.262 (0.199–0.344)	**<0.001**	0.271 (0.192–0.381)	**<0.001**
BEX5	695				
Low	348	Reference			
High	347	0.993 (0.783–1.260)	0.956		
BEX1	695				
Low	348	Reference			
High	347	0.363 (0.281–0.470)	**<0.001**	0.530 (0.384–0.732)	**<0.001**

GBM, glioblastoma multiforme. The bold values were considered statistically significant.

### Correlation Between BEX Family Expression and Immune Infiltration

Immune infiltration analysis showed that BEX family expression was negatively correlated with the infiltration of Th cell, T cells, Tregs, B cells, mast cells, macrophage, neutrophils, cytotoxic cells, Th cells, Th17 cells, Tem, DCs, eosinophils, NK CD56dim, CD8 T cells, and iDCs and was positively correlated with the abundance of NK CD56bright cells and Tgd, Treg, Tfh, and pDC (**Supplementary Figure 2**).

### Functional Enrichment Analysis of BEX Family Genes and Their Differentially Expressed Genes in Glioblastoma Multiforme

#### Identification of Differentially Expressed Genes Between the High and Low BEX Expression Groups

The data from TCGA were analyzed using the DSEeq2 package in R (|log FC| > 1.5, adjusted p-value <0.05), and 100 DEGs (protein-coding genes) were identified between the groups with high and low BEX family expression groups (20 top DEGs for each BEX family member) (shown in **Supplementary Table 2**).

#### Functional Annotation and Enrichment Analysis

To better understand the functional implication of BEX family members in GBM from the top 100 DEGs identified between the high and low expression groups, GO term analysis and KEGG pathway enrichment analysis were performed. The results suggested that BEX family genes and their DEGs were mainly involved in the humoral immune response, B cell-mediated immunity, lymphocyte-mediated immunity, lymphocyte-mediated immunity, immunoglobulin complex related terms, and immunoglobulin receptor binding process, which are immune response-related biological processes and pathways (**Supplementary Figure 3**).

**Figure 3:**
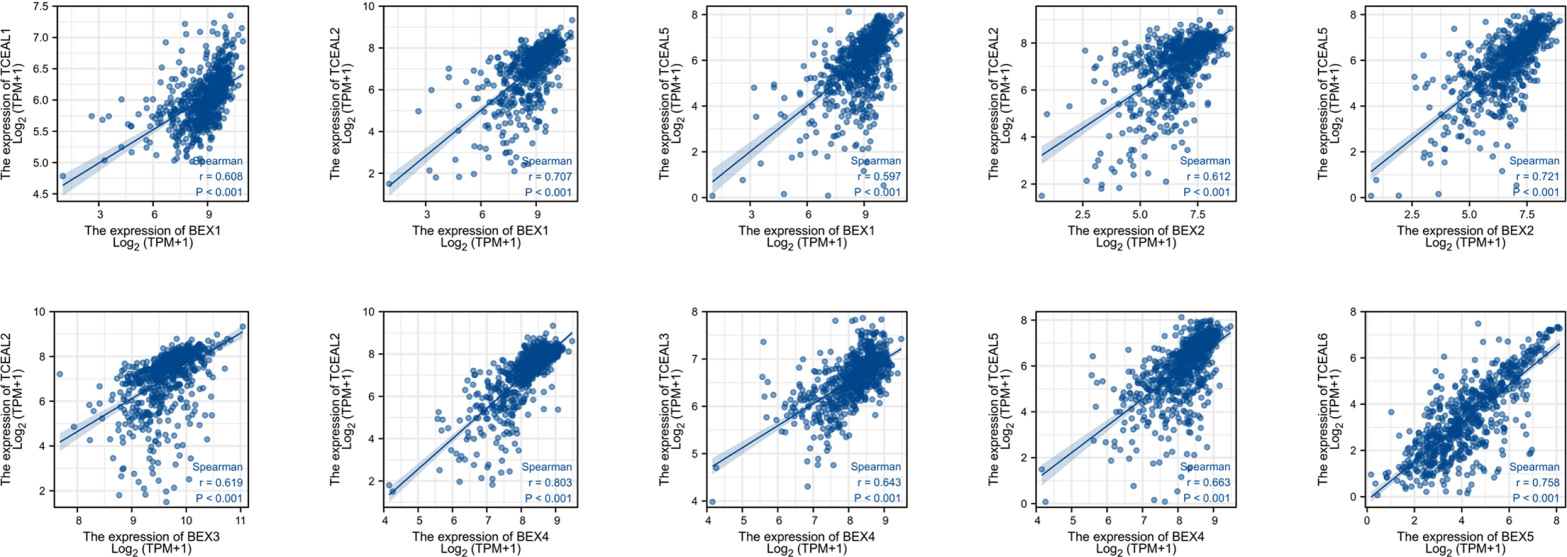
Single-gene correlation analysis showed that BEX family was highly correlated with TCEAL family.

We screened the top 20 genes with the strongest correlation with each BEX family member through stat package Pearson analysis and established a protein interaction network using the string database. The results showed that there was a close and complex correlation between BEX family genes and TCEAL family genes (**Supplementary Figure 4**). Single-gene correlation analysis, once again, verified that there was a significant correlation between the expression levels of BEX family genes and TCEAL family genes in GBM (**Table 2** and **Figure 3**).

**Table 2:** There was a significant correlation between the expression levels of BEX family genes and TCEAL family genes in GBM (Pearson’s analysis).

BEX family	TCEAL family	Cor_ Pearson
BEX2	TCEAL5	0.701584
BEX2	TCEAL6	0.59547
BEX2	TCEAL2	0.595103
BEX3	TCEAL4	0.562
BEX3	TCEAL3	0.532
BEX3	TCEAL5	0.489
BEX3	TCEAL2	0.483
BEX3	TCEAL1	0.48
BEX4	TCEAL2	0.785
BEX5	TCEAL6	0.782218

GBM, glioblastoma multiforme.

#### Expression Difference of TCEAL Family in Glioblastoma Multiforme and Its Correlation With Prognosis

**Figure 4:**
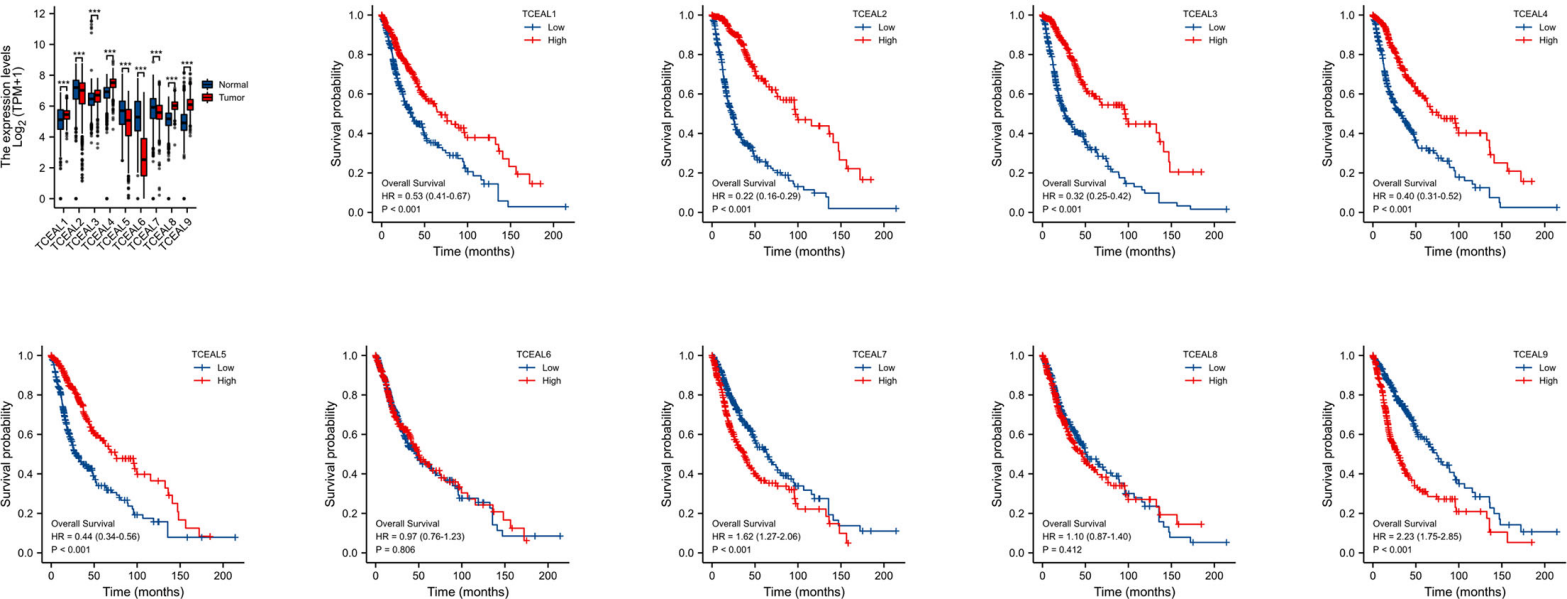
mRNA expression of TCEAL family in GBM and the prognostic value of TCEAL family mRNA expression for OS in GBM patients by Kaplan–Meier analysis. GBM, glioblastoma multiforme; OS, overall survival ns, p≥0.05; *p< 0.05; **p<0.01; ***p<0.001.

According to the correlation analysis of TCGA database, compared with normal tissues, the mRNA expression of TCEAL1, 3, 4, 8, and 9 in GBM was significantly upregulated, and the mRNA expression of TCEAL2, 5, 6, and 7 was significantly downregulated. In addition, Kaplan–Meier survival curves based on median expression values showed that high expression of TCEAL1~5 and TCEAL7 and 9 had better prognosis in OS (log-rank p-score <0.001), while TCEAL6 and TCEAL8 had no significant prognostic value (**Figure 4**). After univariate Cox regression analysis, we found that TCEAL1~5 and TCEAL7 and 9 had a significant prognostic correlation with OS. Multivariate Cox regression analysis showed that TCEAL2 (HR: 0.322; p < 0.001), TCEAL4 (HR: 0.690; p = 0.029), TCEAL7 (HR: 1.873; p < 0.001), and TCEAL9 (HR: 1.913; p < 0.001) had an independent prognostic value for GBM (**Supplementary Table 3**).

## Discussion

### Aberrant Expression of BEX Family Genes in Glioblastoma Multiforme Samples

Many studies have reported that the abnormal expression of BEX family genes was related to the development and prognosis of tumors, such as GBM, gastric cancer, colorectal cancer, hepatocellular carcinoma, lung cancer, and breast cancer (11, 25–28). Here, through the analysis of GBM samples in TCGA database, we once again verify that, except for BEX3, BEX family genes were significantly downregulated in GBM. We also found that there were significant differences in the expression level of BEX family genes among different WHO-G stages, pathological types, and IDH status (except BEX5), and the expression of BEX was lower in the group with higher malignancy and worse prognosis. We also searched for some information about immunohistochemical staining of the BEX family in GBM patients in THE HUMAN PROTEIN ATLAS database (https://www.proteinatlas.org/) and found that the immunohistochemical staining of the BEX family genes performed significantly differently between normal brain tissues and GBM tissues of different grades (**Supplementary Figure 7**). This supported our results that BEX family genes did express differently in GBM patients compared with normal tissues and have some heterogeneity between different grades. Low-grade GBM is generally stained lower than high-grade GBM (except for BEX3). Therefore, we speculate that BEX family genes play a tumor-suppressive role in GBM as a whole facture.

However, previous studies have suggested the oncogenic or tumor-suppressive roles of BEXs, with much controversy. Foltz et al. reported that BEX1 and BEX2 were silenced in GBM and may play an important role in a novel signaling pathway regulating apoptosis as tumor suppressor genes (13). Yan and his colleagues found that BEX1 expression was decreased in GBM (24). Tan et al. reported that BEX2 negatively modulates the hedgehog signaling pathway by retaining Zic2 in the cytoplasm in colorectal cancer cells, thereby inhibiting migration and metastasis of colorectal cancer cells (26). Another study found that the expression of BEX1 and BEX4 was upregulated in radiotherapy-resistant GBM cells and enhanced the tumor formation, growth, and radioresistance of GBM cells by activating the YAP/TAZ signaling pathway (11). Zhou et al. reported that Bex2 was upregulated in GBM and regulated cell proliferation and apoptosis *via* the c-Jun NH2-terminal kinase pathway (12). These different research conclusions illustrated that further relevant studies are needed to explore the specific mechanism of BEX family genes in the occurrence and development of GBM.

### Identification of Prognosis‐Related BEXs in Glioblastoma Multiforme Samples

Members of the BEX family have also reported a prognostic correlation in other tumors, such as gastric cancer and liver cancer (27, 29). In our study, it can also be found that the downregulation of the BEX genes was correlated with the worse prognosis (except for BEX5). Multivariate Cox regression analysis showed that BEX1, BEX2, and BEX4 were independent risk factors for the prognosis of GBM. However, the specific mechanism of how they affect the prognosis of GBM patients is unknown, and further studies are needed.

### Associations of BEX Family With Immune Infiltration

In our study, the immune infiltration analysis showed that the expression of BEX family genes in GBM was negatively correlated with the infiltration of most of the immune cells. The enrichment analysis of the BEX family and their neighbor DEGs suggested that the BEX family and their DEGs were mainly involved in the immune response-related biological processes and pathways including humoral immune response and lymphocyte-mediated immunity, especially B cell-mediated immunity. To further investigate the functions of the BEX family in GBM, we performed GSEA using TCGA data (**Supplementary Figure 4**). GSEA showed that PD-1 signaling, CTLA4 pathway, DNA methylation, P53 signaling pathway, etc. were differentially enriched in the BEX low expression group. Therefore, it can be reasonably speculated that the abnormal expression of BEX family genes may affect the immune cell infiltration of GBM patients, change the normal immune microenvironment, and promote the occurrence and development of GBM by affecting the signal pathway related to immune response.

### Correlation Between BEX Family and TCEAL Family

The single-gene correlation analysis and PPI network show that there is a close and complex correlation between BEX family genes and TCEAL family genes. The correlation between the two gene families has been reported in previous studies that BEX family proteins and BEX domains are also found in TCEAL proteins (30, 31), and the function of this domain is involved in the control of cellular growth (32–34).

Our study also showed that compared with the normal tissues, the expression of TCEAL1, 3, 4, 8, and 9 was significantly upregulated in GBM, and the expression of TCEAL2, 5, 6, and 7 was significantly downregulated in GBM. In addition, survival analysis and univariate Cox regression analysis showed that patients with high expression of TCEAL1~5 and TCEAL7 and 9 had longer OS. Multivariate Cox regression analysis showed that TCEAL2, TCEAL4, TCEAL7, and TCEAL9 had independent predictive values for the prognosis of GBM. The previous results suggest that BEX1, BEX2, and BEX4 also have an independent prognostic value for GBM. Therefore, we use the above seven genes to establish a preliminary multivariate prognostic model.

### Construction of a Prognostic Scoring Model Based on BEX1, BEX2, BEX4, TCEAL2, TCEAL4, TCEAL7, and TCEAL9 Expression

To provide clinicians with a quantitative approach for predicting the prognosis of GBM patients, a nomogram was constructed that integrated the clinical characteristics as well as BEX and TCEAL members determined to be independently associated with survival *via* multivariate analysis (WHO-G grade, IDH status, and 1p/19q codeletion). Within the nomogram (**Supplementary Figure 5A**), WHO-G grade was found to contribute the most extreme data points (ranging from 0 to 100) as compared with the other clinical variables, which was consistent with the results of multivariate Cox regression. The C-index of the nomogram (**Supplementary Figure 5B**) was 0.836 (95% CI: 0.824–0.849). The bias-corrected line in the calibration plot was close to the ideal curve, indicating a good agreement between the predicted and observed values. Overall, the nomogram was found to be a superior model for predicting long-term survival in GBM patients than individual prognostic factors.

**Figure 5:**
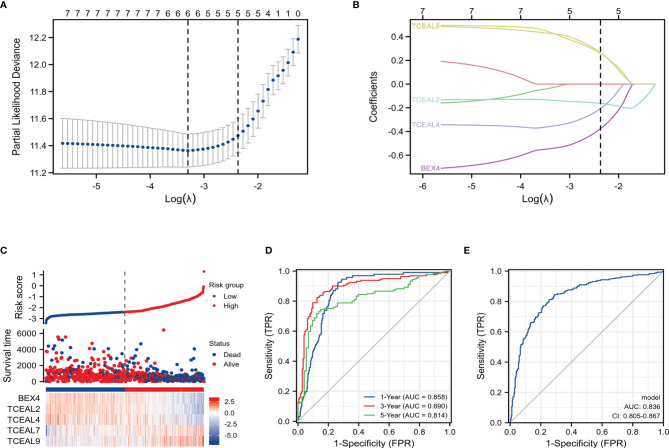
LASSO Cox regression model of GBM and ROC curves analysis of LASSO Cox regression model. **(A)** Partial likelihood deviance of OS for the LASSO coefficient profiles. **(B)** LASSO coefficient profiles of the BEX4 and TCEAL2, TCEAL4, TCEAL7, and TCEAL9 expression for OS. **(C)** The survival time of GBM patients in the high-risk group was significantly shorter than that of patients in the low-risk group. **(D)** The time-dependent ROC curve; the AUC values of 1-, 3-, and 5-year OS were 0.858, 0.890, and 0.814, respectively. **(E)** The AUC value of the diagnostic ROC curve was 0.836. LASSO, least absolute shrinkage and selection operator; GBM, glioblastoma multiforme; ROC, receiver operating characteristic; OS, overall survival; AUC, area under the curve.

In order to construct a risk score model for predicting OS of GBM, the LASSO Cox regression model was used to build a prognostic classifier, which included BEX1, BEX2, BEX4, sf5TCEAL2, TCEAL4, TCEAL7, and TCEAL9 (**Figures 5A,B**). Using the LASSO Cox regression models, we calculated a risk score for each patient: risk score = (BEX4 * −0.3762202) + (TCEAL2 * −0.1629317) + (TCEAL4 * −0.2063245) + (TCEAL7 * 0.25805615) + (TCEAL9 * 0.26348946). Survival analysis revealed that the survival time of GBM patients in the high-risk group was significantly shorter than that of patients in the low-risk group (**Figure 5C**). Then, the model reliability was verified through the ROC curves analysis. In the time-dependent ROC curve, the AUC values of 1-, 3- and 5-year OS were 0.858, 0.890 and 0.814, respectively (**Figure 5D**), and the AUC value of the diagnostic ROC curve was 0.836 (**Figure 5E**). The above results indicated that the BEX and TCEAL risk assessment models had a predictive value for the prognosis of GBM patients.

In the present study, there still exist some limitations. First, we acknowledge that, in this study, we did not rigorously test whether the proportional hazards assumption holds. To furtherly confirm the assumption of proportional hazards, time-dependent covariate analysis could be suggested, and if the premise of proportional hazards does not hold, it is generally recommended to use the limited mean survival time instead of the median survival time for the description and comparison of survival analysis. Second, our present study is limited to the existing information in TCGA database, and more in-depth studies are necessary to verify the results with an expanded sample size in the future. What is more, the mechanisms of the BEX family involved in the initiation and progression of GBM, especially in the immune regulation processes, require further investigation.

Taken together, by assessing the global gene expression profile, we demonstrated that except for BEX3, the expression of the other four members of BEX family genes was downregulated in GBM, related to a worse prognosis of GBM, and involved in the initiation and progression of GBM, especially in the immune regulation processes. Among them, BEX1, 2, and 4 were independently correlated with the prognosis of GBM. In addition, there is a significant correlation between BEX family genes and TCEAL family genes, which also had an abnormal expression in GBM, and were significantly related to the prognosis of GBM patients. Based on the above conclusions, we established an accurate and practical prognosis prediction model with the two gene families and clinical characteristics independently related to the prognosis of GBM.

## Data Availability Statement

The datasets presented in this study can be found in online repositories. The names of the repository/repositories and accession number(s) can be found in the article/**Supplementary Material**.

## Author Contributions

AA, YT, YS, and YY: conceptualization. AA: methodology. AA, DZ, and XL: software. AA and YT: validation. AA and XL: formal analysis. DZ and XL: data curation. AA, DZ, and XL: writing—original draft preparation. YT, YS, and YY: writing—review and editing. All authors contributed to the article and approved the submitted version.

## Funding

This research was supported by the National Natural Science Foundation of China (82102708 to YT), the Zhejiang Provincial Natural Science Foundation of China under Grant (LQ21H160024 to YT), the Zhejiang Provincial Medicine and Technology Projects Grant (2020RC063 to YT), the Zhejiang Provincial Key R&D Program (2021C03125 to YY), and the Health Commission of Zhejiang Province (grant number: 2019KY082).

## Conflict of Interest

The authors declare that the research was conducted in the absence of any commercial or financial relationships that could be construed as a potential conflict of interest.

## Publisher’s Note

All claims expressed in this article are solely those of the authors and do not necessarily represent those of their affiliated organizations, or those of the publisher, the editors and the reviewers. Any product that may be evaluated in this article, or claim that may be made by its manufacturer, is not guaranteed or endorsed by the publisher.

## Supplementary Material

Supplementary Figure 1 Relationship between BEX family expression and clinical indicators in GBM patients. **(A)** Correlation between gene expression level of BEX family and WHO-G stage in glioma patients. **(B)** Correlation between gene expression level of BEX family and IDH status in glioma patients. **(C)** Correlation between gene expression level of BEX family and 1p19q codeletion in glioma patients. **(D)** Correlation between gene expression level of BEX family and histological type in glioma patients.

Supplementary Figure 2 Immune infiltration analysis of BEX family in GBM. Immune cells. aDC [activated DC]; B cells; CD8 T cells; Cytotoxic cells; DC; Eosinophils; iDC [immature DC]; Macrophages; Mast cells; Neutrophils; NK CD56bright cells; NK CD56dim cells; NK cells; pDC [Plasmacytoid DC]; T cells; T helper cells; Tcm [T central memory]; Tem [T effector memory]; Tfh [T follicular helper]; Tgd [T gamma delta]; Th1 cells; Th17 cells; Th2 cells; Treg.

Supplementary Figure 3 GO term analysis and KEGG pathway enrichment analysis of BEX family in GBM.

Supplementary Figure 4 PPI network of BEX family and TCEAL family: there was a significant correlation between the expression levels of BEX family genes and TCEAL family genes in GBM.

Supplementary Figure 5 Enrichment plots from gene set enrichment analysis (GSEA). PD-1signaling, CTLA4 pathway, DNA methylation and P53 signaling pathway et al. were enriched in low BEXs expression phenotypes and might be closely correlated with prognosis of GBM.

Supplementary Figure 6 Relationship between 7 Independent prognostic value genes and other clinical factors with overall survival (OS). **(A)** Nomogram for predicting the probability of 1-, 3-, and 5-year OS for GBM patients. **(B)** Calibration plot of the nomogram for predicting the OS likelihood. (Concordance, C-index: 0.836; 95% confidence interval:0.824-0.849).

Supplementary Figure 7 The immunohistochemical staining of the BEX family genes in normal brain tissues and GBM tissues of different grades: BEX family genes generally stained medium to high in brain tissues, especially in neuronal cells, when they stained medium to low in high grade GBM and stained low or not detected in low grade GBM. (except for BEX3,which stained high in low grade GBM).
