# Supplementary figures and images for: Comprehensive Analysis of the Brain-Expressed X-Link Protein Family in Glioblastoma Multiforme
Source: Front Oncol. 2022 Jul 4;12:911942. doi: 10.3389/fonc.2022.911942 (PMC9289282; doi:10.3389/fonc.2022.911942)

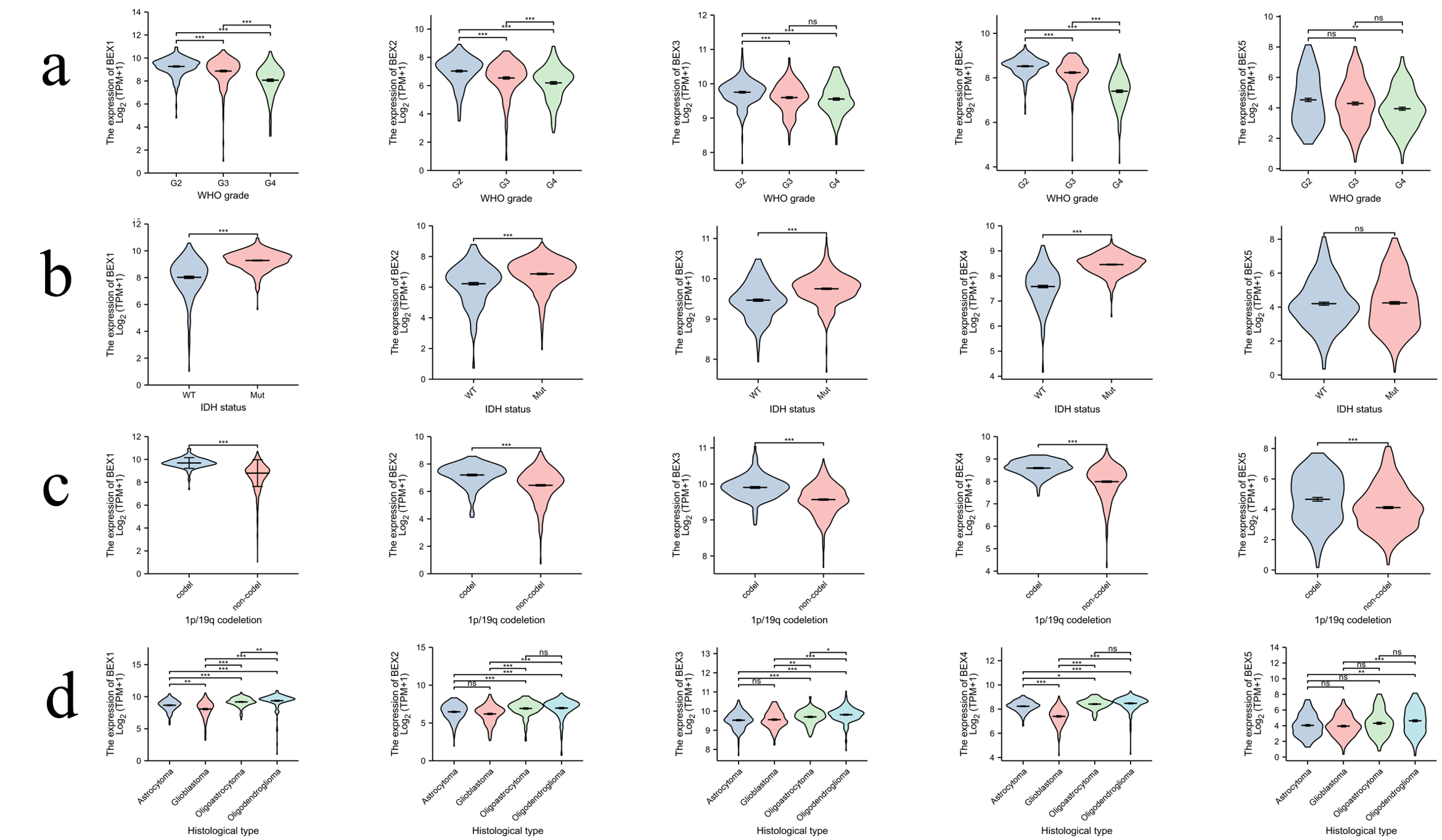

Supplement: Supplementary Figure 1 — Relationship between BEX family expression and clinical indicators in GBM patients. (A) Correlation between gene expression level of BEX family and WHO-G stage in glioma patients. (B) Correlation between gene expression level of BEX family and IDH status in glioma patients. (C) Correlation between gene expression level of BEX family and 1p19q codeletion in glioma patients. (D) Correlation between gene expression level of BEX family and histological type in glioma patients. [file Image_1.tif]

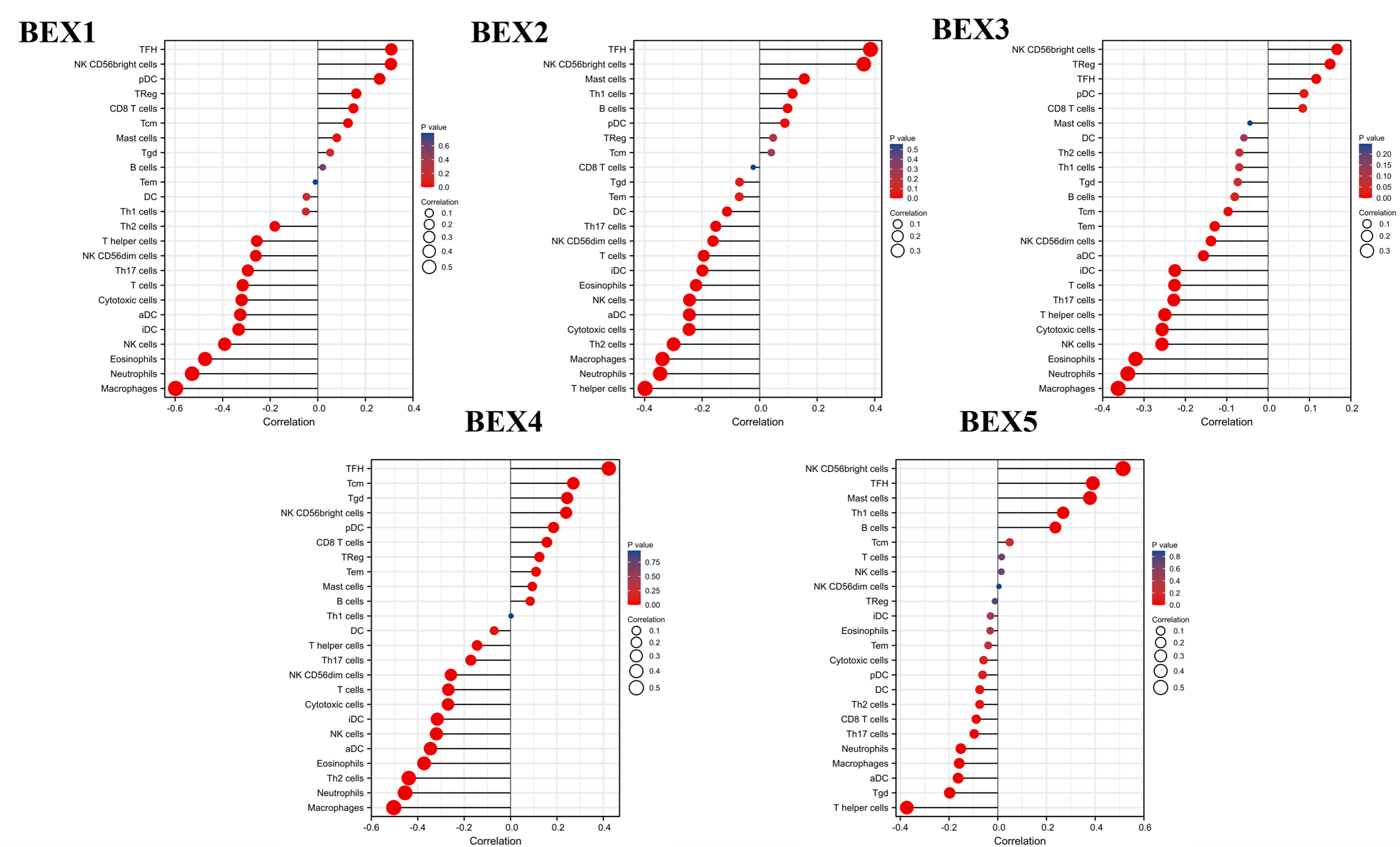

Supplement: Supplementary Figure 2 — Immune infiltration analysis of BEX family in GBM. Immune cells. aDC [activated DC]; B cells; CD8 T cells; Cytotoxic cells; DC; Eosinophils; iDC [immature DC]; Macrophages; Mast cells; Neutrophils; NK CD56bright cells; NK CD56dim cells; NK cells; pDC [Plasmacytoid DC]; T cells; T helper cells; Tcm [T central memory]; Tem [T effector memory]; Tfh [T follicular helper]; Tgd [T gamma delta]; Th1 cells; Th17 cells; Th2 cells; Treg. [file Image_2.tif]

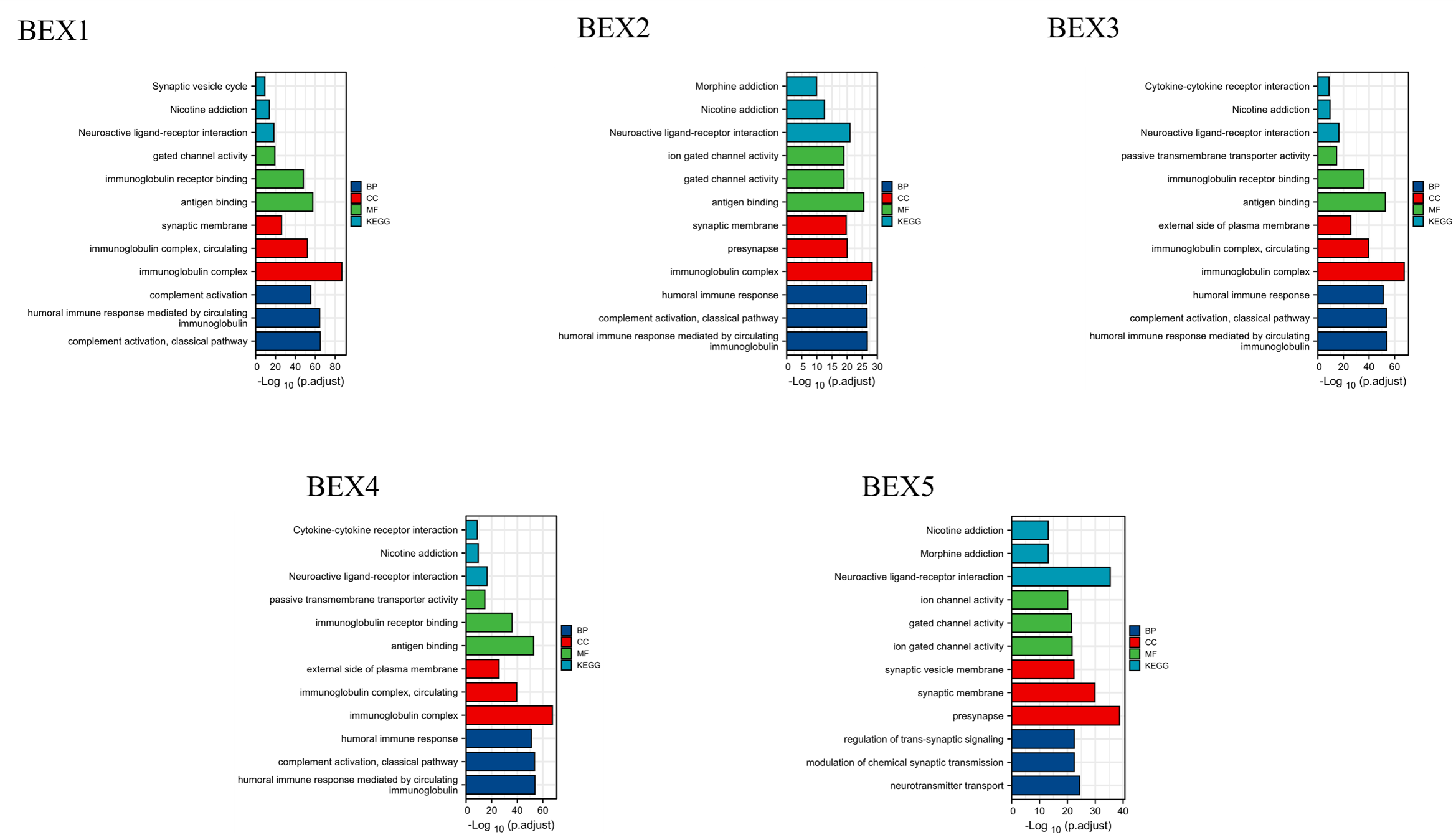

Supplement: Supplementary Figure 3 — GO term analysis and KEGG pathway enrichment analysis of BEX family in GBM. [file Image_3.tif]

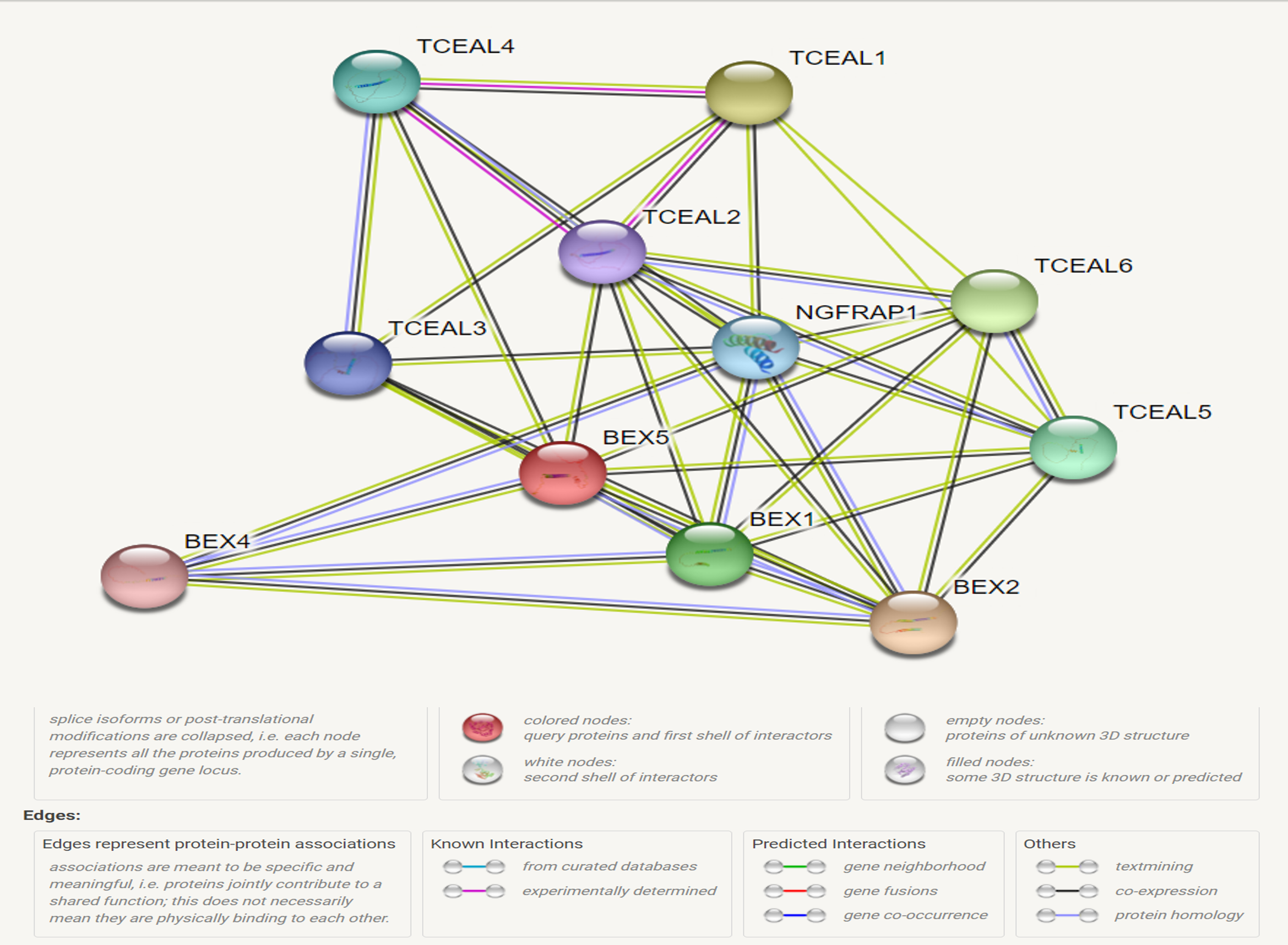

Supplement: Supplementary Figure 4 — PPI network of BEX family and TCEAL family: there was a significant correlation between the expression levels of BEX family genes and TCEAL family genes in GBM. [file Image_4.tif]

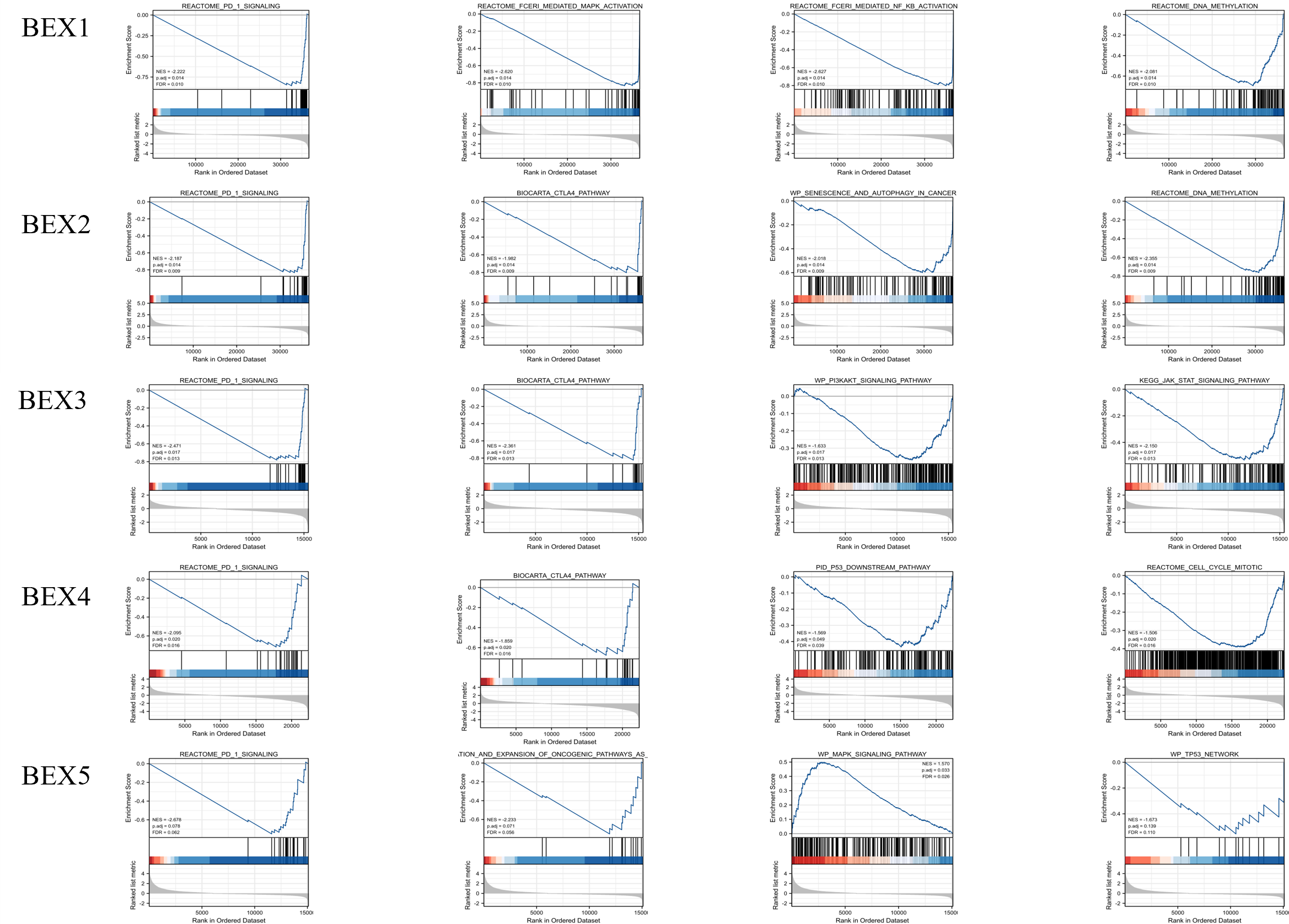

Supplement: Supplementary Figure 5 — Enrichment plots from gene set enrichment analysis (GSEA). PD-1signaling, CTLA4 pathway, DNA methylation and P53 signaling pathway et al. were enriched in low BEXs expression phenotypes and might be closely correlated with prognosis of GBM. [file Image_5.tif]

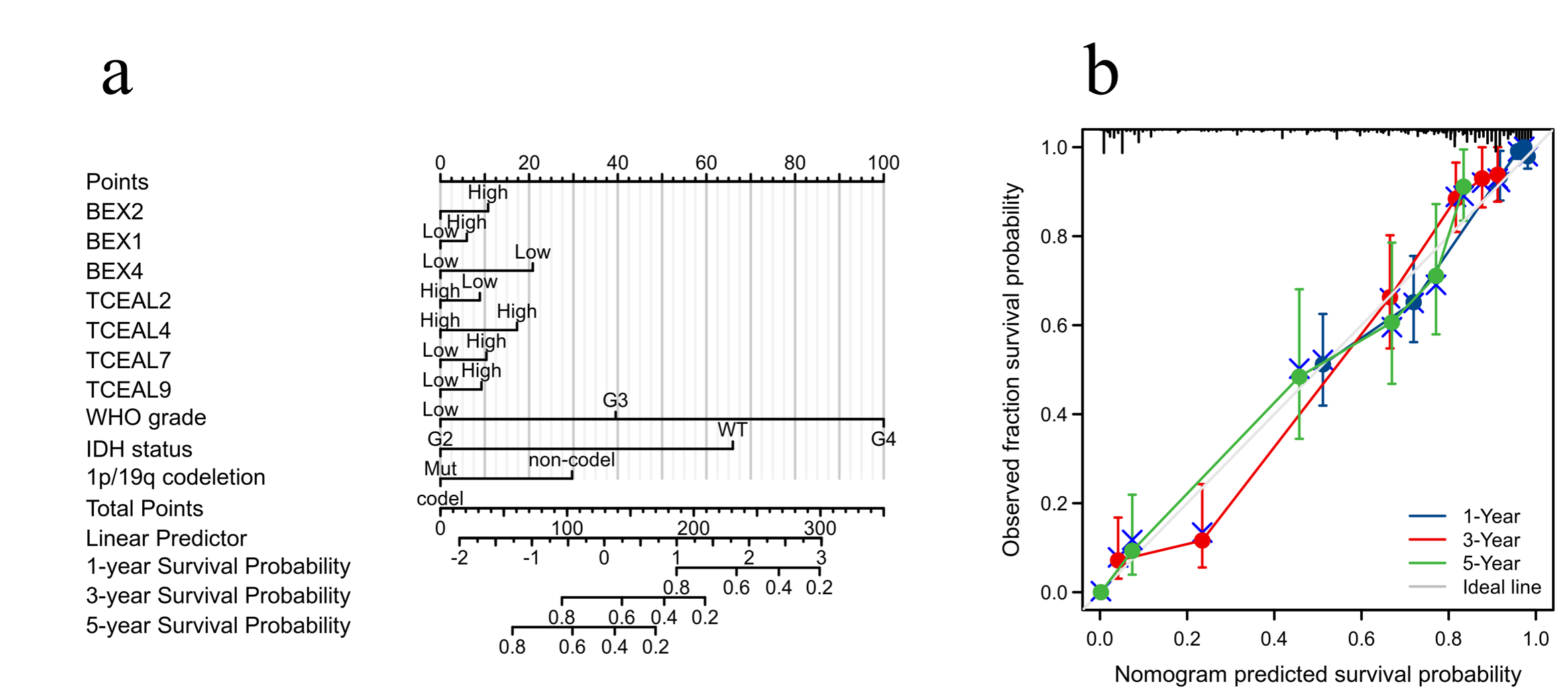

Supplement: Supplementary Figure 6 — Relationship between 7 Independent prognostic value genes and other clinical factors with overall survival (OS). (A) Nomogram for predicting the probability of 1-, 3-, and 5-year OS for GBM patients. (B) Calibration plot of the nomogram for predicting the OS likelihood. (Concordance, C-index: 0.836; 95% confidence interval:0.824-0.849). [file Image_6.tif]

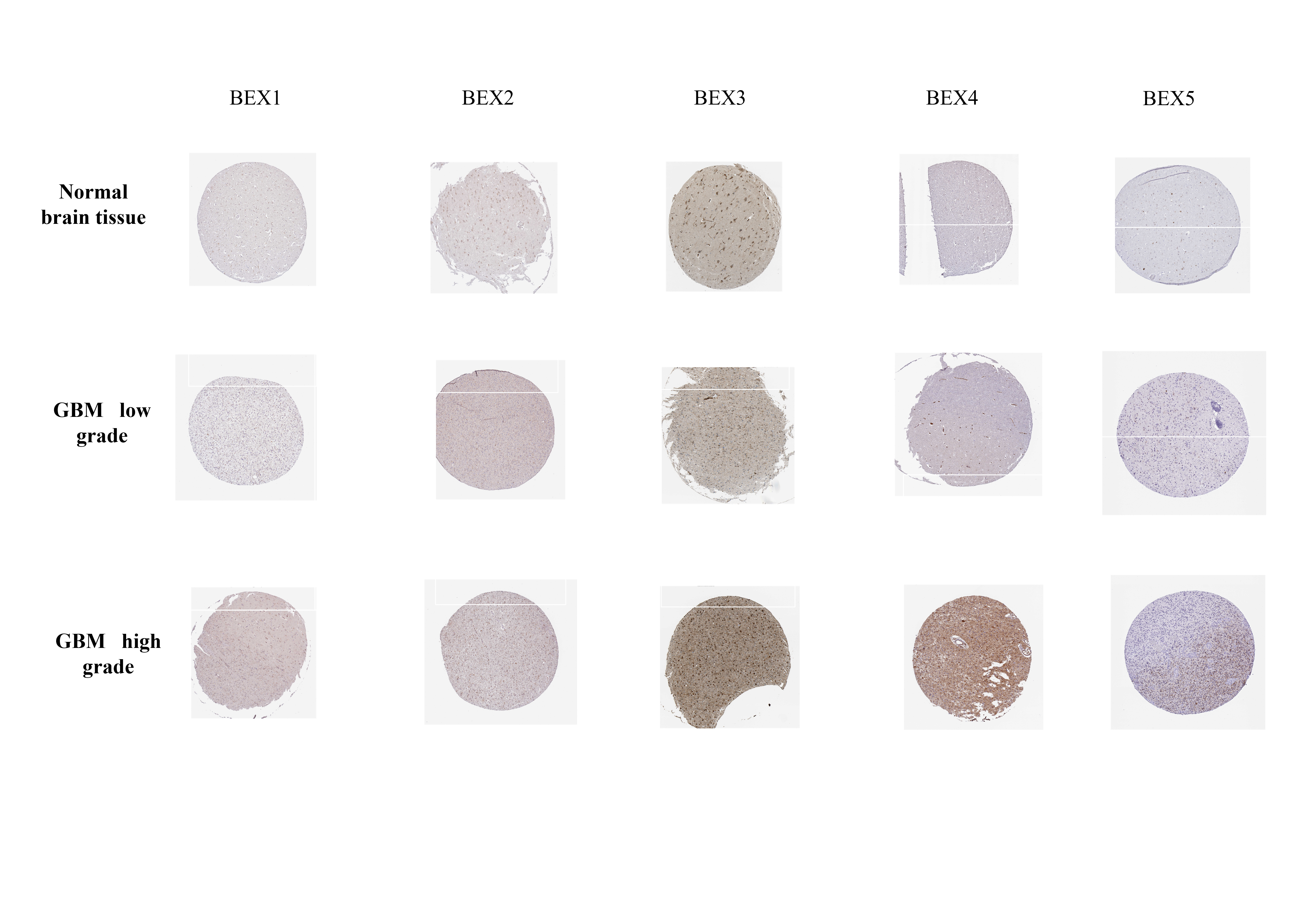

Supplement: Supplementary Figure 7 — The immunohistochemical staining of the BEX family genes in normal brain tissues and GBM tissues of different grades: BEX family genes generally stained medium to high in brain tissues, especially in neuronal cells, when they stained medium to low in high grade GBM and stained low or not detected in low grade GBM. (except for BEX3,which stained high in low grade GBM). [file Image_7.tif]
