# Supplementary material for: Comprehensive Analysis of the Brain-Expressed X-Link Protein Family in Glioblastoma Multiforme
Source: Front Oncol. 2022 Jul 4;12:911942. doi: 10.3389/fonc.2022.911942 (PMC9289282; doi:10.3389/fonc.2022.911942)
Supplement: Supplementary file 8 [file Table_1.docx]

Supplementary Table1: Relationship between BEX family expression and clinical parameters of GBM patients using the TCGA database

|  | Characteristic | Low BEX1 | High BEX1 | p | Low BEX2 | High BEX2 | p | Low BEX3 | High BEX3 | p | Low BEX4 | High BEX4 | p | Low BEX5 | High BEX5 | p |  |
| --- | --- | --- | --- | --- | --- | --- | --- | --- | --- | --- | --- | --- | --- | --- | --- | --- | --- |
|  | n | 348 | 348 |  | 348 | 348 |  | 348 | 348 |  | 348 | 348 |  | 348 | 348 |  |  |
|  | WHO grade,  n (%) |  |  | < 0.001 |  |  | < 0.001 |  |  | < 0.001 |  |  | < 0.001 |  |  | 0.033 |  |
|  | G2 | 73 (11.5%) | 151  (23.8%) |  | 73 (11.5%) | 151 (23.8%) |  | 83 (13.1%) | 141 (22.2%) |  | 54 (8.5%) | 170 (26.8%) |  | 104 (16.4%) | 120 (18.9%) |  |  |
|  | G3 | 118 (18.6%) | 125  (19.7%) |  | 132 (20.8%) | 111 (17.5%) |  | 135 (21.3%) | 108  (17%) |  | 115 (18.1%) | 128 (20.2%) |  | 111 (17.5%) | 132 (20.8%) |  |  |
|  | G4 | 134 (21.1%) | 34  (5.4%) |  | 118 (18.6%) | 50 (7.9%) |  | 101 (15.9%) | 67  (10.6%) |  | 155 (24.4%) | 13  (2%) |  | 97 (15.3%) | 71  (11.2%) |  |  |
|  | IDH status, n (%) |  |  | < 0.001 |  |  | < 0.001 |  |  | < 0.001 |  |  | < 0.001 |  |  | 0.901 |  |
|  | WT | 193 (28.1%) | 53  (7.7%) |  | 162 (23.6%) | 84 (12.2%) |  | 169 (24.6%) | 77  (11.2%) |  | 207 (30.2%) | 39  (5.7%) |  | 125 (18.2%) | 121 (17.6%) |  |  |
|  | Mut | 146 (21.3%) | 294  (42.9%) |  | 180 (26.2%) | 260 (37.9%) |  | 174 (25.4%) | 266 (38.8%) |  | 133 (19.4%) | 307 (44.8%) |  | 220 (32.1%) | 220 (32.1%) |  |  |
|  | 1p/19q codeletion, n (%) |  |  | < 0.001 |  |  | < 0.001 |  |  | < 0.001 |  |  | < 0.001 |  |  | 0.001 |  |
|  | codel | 8  (1.2%) | 163  (23.7%) |  | 43 (6.2%) | 128 (18.6%) |  | 38 (5.5%) | 133 (19.3%) |  | 30 (4.4%) | 141 (20.5%) |  | 66 (9.6%) | 105 (15.2%) |  |  |
|  | non-codel | 334 (48.5%) | 184  (26.7%) |  | 299 (43.4%) | 219 (31.8%) |  | 306 (44.4%) | 212 (30.8%) |  | 311 (45.1%) | 207  (30%) |  | 276 (40.1%) | 242 (35.1%) |  |  |
|  | Histological type, n (%) |  |  | < 0.001 |  |  | < 0.001 |  |  | < 0.001 |  |  | < 0.001 |  |  | < 0.001 |  |
|  | Astrocytoma | 131 (18.8%) | 64  (9.2%) |  | 116 (16.7%) | 79 (11.4%) |  | 128 (18.4%) | 67  (9.6%) |  | 97 (13.9%) | 98 (14.1%) |  | 110 (15.8%) | 85  (12.2%) |  |  |
|  | Glioblastoma | 134 (19.3%) | 34  (4.9%) |  | 118 (17%) | 50 (7.2%) |  | 101 (14.5%) | 67  (9.6%) |  | 155 (22.3%) | 13  (1.9%) |  | 97 (13.9%) | 71  (10.2%) |  |  |
|  | Oligoastrocytoma | 48  (6.9%) | 86  (12.4%) |  | 51 (7.3%) | 83 (11.9%) |  | 57 (8.2%) | 77  (11.1%) |  | 44 (6.3%) | 90 (12.9%) |  | 62 (8.9%) | 72  (10.3%) |  |  |
|  | Oligodendroglioma | 35  (5%) | 164  (23.6%) |  | 63 (9.1%) | 136 (19.5%) |  | 62 (8.9%) | 137 (19.7%) |  | 52 (7.5%) | 147 (21.1%) |  | 79 (11.4%) | 120 (17.2%) |  |  |
|  | OS event, n (%) |  |  | < 0.001 |  |  | < 0.001 |  |  | < 0.001 |  |  | < 0.001 |  |  | 0.816 |  |
|  | Alive | 168 (24.1%) | 256  (36.8%) |  | 183 (26.3%) | 241 (34.6%) |  | 179 (25.7%) | 245 (35.2%) |  | 148 (21.3%) | 276 (39.7%) |  | 210 (30.2%) | 214 (30.7%) |  |  |
|  | Dead | 180 (25.9%) | 92  (13.2%) |  | 165 (23.7%) | 107 (15.4%) |  | 169 (24.3%) | 103 (14.8%) |  | 200 (28.7%) | 72 (10.3%) |  | 138 (19.8%) | 134 (19.3%) |  |  |
|  | Gender, n (%) |  |  | 0.146 |  |  | 0.078 |  |  | 0.055 |  |  | 0.055 |  |  | 0.939 |  |
|  | Female | 139 (20%) | 159  (22.8%) |  | 137 (19.7%) | 161 (23.1%) |  | 136 (19.5%) | 162 (23.3%) |  | 136 (19.5%) | 162 (23.3%) |  | 148 (21.3%) | 150 (21.6%) |  |  |
|  | Male | 209 (30%) | 189  (27.2%) |  | 211 (30.3%) | 187 (26.9%) |  | 212 (30.5%) | 186 (26.7%) |  | 212 (30.5%) | 186 (26.7%) |  | 200 (28.7%) | 198 (28.4%) |  |  |
|  | Age, n (%) |  |  | < 0.001 |  |  | < 0.001 |  |  | < 0.001 |  |  | < 0.001 |  |  | 0.851 |  |
|  | <=60 | 244 (35.1%) | 309  (44.4%) |  | 254 (36.5%) | 299 (43%) |  | 255 (36.6%) | 298 (42.8%) |  | 240 (34.5%) | 313  (45%) |  | 275 (39.5%) | 278 (39.9%) |  |  |
|  | >60 | 104 (14.9%) | 39  (5.6%) |  | 94 (13.5%) | 49  (7%) |  | 93 (13.4%) | 50  (7.2%) |  | 108 (15.5%) | 35  (5%) |  | 73 (10.5%) | 70  (10.1%) |  |  |
