# Supplementary material for: Comprehensive Analysis of the Brain-Expressed X-Link Protein Family in Glioblastoma Multiforme
Source: Front Oncol. 2022 Jul 4;12:911942. doi: 10.3389/fonc.2022.911942 (PMC9289282; doi:10.3389/fonc.2022.911942)
Supplement: Supplementary file 9 [file Table_2.docx]

Supplementary Table 2：100 DEGs (protein-coding genes) between the groups with high and low BEX family expression.

|  | BEX1 | |  | BEX2 | |  | BEX3 | |  | BEX4 | |  | BEX5 | |  |
| --- | --- | --- | --- | --- | --- | --- | --- | --- | --- | --- | --- | --- | --- | --- | --- |
|  | gene | cor_pearson |  | gene | cor_pearson |  | gene | cor_pearson |  | gene | cor_pearson |  | gene | cor_pearson |  |
|  | GDAP1L1 | 0.806819 |  | TCEAL5 | 0.701584 |  | KLHDC3 | 0.596 |  | TCEAL2 | 0.78498 |  | TCEAL6 | 0.782218 |  |
|  | RUNDC3A | 0.803618 |  | BEX1 | 0.686182 |  | BEX1 | 0.595 |  | NAP1L3 | 0.779364 |  | CACNG3 | 0.771127 |  |
|  | CELF3 | 0.750949 |  | BEX4 | 0.684292 |  | EIPR1 | 0.591 |  | SNAP91 | 0.744701 |  | GABRA1 | 0.768535 |  |
|  | NSG2 | 0.748598 |  | HPCAL4 | 0.659785 |  | CHCHD6 | 0.590 |  | KCNB1 | 0.741335 |  | SLC6A7 | 0.767631 |  |
|  | ACTL6B | 0.748299 |  | SYP | 0.644786 |  | GLRX5 | 0.563 |  | SH3GL2 | 0.738315 |  | MAL2 | 0.767366 |  |
|  | ATCAY | 0.746658 |  | SYN1 | 0.643322 |  | TCEAL4 | 0.562 |  | ATP6V1G2 | 0.736811 |  | SULT4A1 | 0.767348 |  |
|  | TLCD3B | 0.744051 |  | TAGLN3 | 0.640774 |  | CUEDC2 | 0.560 |  | NAP1L2 | 0.729206 |  | GABRB2 | 0.76551 |  |
|  | MAPK8IP2 | 0.74329 |  | CHRM1 | 0.63987 |  | GDI1 | 0.558 |  | RTN1 | 0.726652 |  | MAP7D2 | 0.765058 |  |
|  | SERP2 | 0.737407 |  | SNAP25 | 0.635181 |  | GPKOW | 0.555 |  | ARPP21 | 0.718066 |  | HTR5A-AS1 | 0.76442 |  |
|  | RIPPLY2 | 0.733162 |  | CAMKV | 0.626335 |  | RNF187 | 0.549 |  | CPEB3 | 0.715296 |  | PHF24 | 0.764225 |  |
|  | SYP | 0.732958 |  | NAP1L2 | 0.618353 |  | IDH3G | 0.544 |  | RUNDC3A | 0.714522 |  | PRSS3 | 0.763721 |  |
|  | FBLL1 | 0.731093 |  | SYT4 | 0.617908 |  | OTUD5 | 0.542 |  | FAIM2 | 0.708278 |  | TMEM130 | 0.761571 |  |
|  | CHGB | 0.727753 |  | RUNDC3A | 0.616121 |  | MRFAP1 | 0.534 |  | JPH4 | 0.704832 |  | HPCA | 0.758991 |  |
|  | SCAMP5 | 0.727676 |  | MAPK8IP2 | 0.612072 |  | TCEAL3 | 0.532 |  | DNAJC12 | 0.703916 |  | SV2B | 0.757565 |  |
|  | JPH4 | 0.726086 |  | CPLX2 | 0.611289 |  | LRRC20 | 0.530 |  | GABBR1 | 0.7038 |  | GNG3 | 0.756741 |  |
|  | SMIM18 | 0.722827 |  | SCN3B | 0.607694 |  | PQBP1 | 0.521 |  | NAP1L5 | 0.703607 |  | GPR22 | 0.756565 |  |
|  | CHRNB2 | 0.722154 |  | TMEM151B | 0.607343 |  | PJA1 | 0.513 |  | OMG | 0.702187 |  | VSNL1 | 0.755694 |  |
|  | ATP6V1G2 | 0.720339 |  | RAB3C | 0.606389 |  | PPP1R7 | 0.510 |  | KCNJ9 | 0.702066 |  | SSTR3 | 0.754619 |  |
|  | MAP2 | 0.713047 |  | CHGA | 0.604405 |  | USP11 | 0.509 |  | NALCN | 0.700512 |  | SLC12A5 | 0.753858 |  |
|  | FXYD6 | 0.712826 |  | SLC8A2 | 0.602992 |  | BEX4 | 0.507 |  | BEX1 | 0.699859 |  | DOC2A | 0.753694 |  |
