# Supplementary material for: Comprehensive Analysis of the Brain-Expressed X-Link Protein Family in Glioblastoma Multiforme
Source: Front Oncol. 2022 Jul 4;12:911942. doi: 10.3389/fonc.2022.911942 (PMC9289282; doi:10.3389/fonc.2022.911942)
Supplement: Supplementary file 10 [file Table_3.docx]

Supplementary Table 3: Univariate and multivariate Cox regression analysis of TCEAL family in GBM

| Characteristics | Total(N) | Univariate analysis | |  | Multivariate analysis | |
| --- | --- | --- | --- | --- | --- | --- |
|  |  | Hazard ratio (95% CI) | P value |  | Hazard ratio (95% CI) | P value |
| TCEAL1 | 695 |  |  |  |  |  |
| Low | 348 | Reference |  |  |  |  |
| High | 347 | 0.526 (0.411-0.673) | **<0.001** |  | 0.796 (0.598-1.058) | 0.116 |
| TCEAL2 | 695 |  |  |  |  |  |
| Low | 348 | Reference |  |  |  |  |
| High | 347 | 0.217 (0.164-0.287) | **<0.001** |  | 0.322 (0.221-0.470) | **<0.001** |
| TCEAL3 | 695 |  |  |  |  |  |
| Low | 348 | Reference |  |  |  |  |
| High | 347 | 0.323 (0.249-0.419) | **<0.001** |  | 0.734 (0.525-1.026) | 0.070 |
| TCEAL4 | 695 |  |  |  |  |  |
| Low | 348 | Reference |  |  |  |  |
| High | 347 | 0.402 (0.313-0.518) | **<0.001** |  | 0.690 (0.495-0.962) | **0.029** |
| TCEAL5 | 695 |  |  |  |  |  |
| Low | 348 | Reference |  |  |  |  |
| High | 347 | 0.436 (0.339-0.561) | **<0.001** |  | 0.919 (0.668-1.263) | 0.601 |
| TCEAL6 | 695 |  |  |  |  |  |
| Low | 348 | Reference |  |  |  |  |
| High | 347 | 0.971 (0.765-1.232) | 0.806 |  |  |  |
| TCEAL7 | 695 |  |  |  |  |  |
| Low | 347 | Reference |  |  |  |  |
| High | 348 | 1.619 (1.273-2.059) | **<0.001** |  | 1.873 (1.421-2.469) | **<0.001** |
| TCEAL8 | 695 |  |  |  |  |  |
| Low | 348 | Reference |  |  |  |  |
| High | 347 | 1.105 (0.871-1.401) | 0.412 |  |  |  |
| TCEAL9 | 695 |  |  |  |  |  |
| Low | 347 | Reference |  |  |  |  |
| High | 348 | 2.234 (1.749-2.854) | **<0.001** |  | 1.913 (1.448-2.528) | **<0.001** |
